# Dietary Behaviors in the Post-Lockdown Period and Its Effects on Dietary Diversity: The Second Stage of a Nutrition Survey in a Longitudinal Chinese Study in the COVID-19 Era

**DOI:** 10.3390/nu12113269

**Published:** 2020-10-26

**Authors:** Jian Zhang, Ai Zhao, Yalei Ke, Shanshan Huo, Yidi Ma, Yumei Zhang, Zhongxia Ren, Zhongyu Li, Keyang Liu

**Affiliations:** 1 Vanke School of Public Health, Tsinghua University, Beijing 100091, China; Jianzhang_bjmu@163.com; 2 School of Public Health, Peking University, Beijing 100191, China; yaleike_bjmu@163.com (Y.K.); shanshanhuo_bjmu@163.com (S.H.); yidima_bjmu@163.com (Y.M.); yumeizhang_bjmu@163.com (Y.Z.); zhongxiaren_bjmu@163.com (Z.R.); 3 Department of International Health, Johns Hopkins Bloomberg School of Public Health, Baltimore, MD 21205, USA; zli132@jhmi.edu; 4 Public Health, Department of Social Medicine, Osaka University Graduate School of Medicine, Osaka 5620032, Japan; liu@pbhel.med.osaka-u.ac.jp

**Keywords:** COVID-19, dietary diversity, post-lockdown, dietary behaviors, China

## Abstract

Coronavirus disease 2019 (COVID-19) has imposed enormous challenges on people’s lifestyles. People in China have gradually returned to normal life; however, in the protracted pandemic, people may still follow certain dietary behaviors to cope with COVID-19. This study was the second stage of a longitudinal nutritional survey conducted in post-lockdown China that was aimed at exploring post-lockdown dietary behaviors and their effects on dietary diversity. In line with the first stage of the survey, the current dietary behaviors used to cope with COVID-19 and ways of purchasing food were determined. In addition, changes in dietary behavior compared to the same period in 2019 and those behaviors recommended to ensure food safety were also investigated. The Household Dietary Diversity Score (HDDS) was used to assess dietary diversity; this was also used in the first stage of the survey. Linear regression was used to model the associations between the HDDS, participants’ characteristics, and dietary behaviors. The data of 1994 participants were included in the analysis. The overall mean HDDS was 9.2 ± 2.0. Compared to the same period in 2019, a substantial proportion of participants self-reported that they had recently decreased eating in restaurants (61.6%) and reduced intakes of seafood (53.1%), imported frozen food (57.1%), and raw food (60.5%), while 64.8% of participants reported increased cooking at home. People with an increased consumption of seafood (adjusted OR (95%CI) = 0.56 (0.07, 1.04)) and raw food (adjusted OR (95%CI) = 0.74 (0.27, 1.21)) had a significantly higher HDDS. Participants who changed their consumption of imported frozen food (both increased and decreased) had a higher HDDS (adjusted OR (95%CI) = 0.56 (0.07, 1.04) and 0.27 (0.09, 0.44), respectively). People who depended more on purchasing food online had a significantly higher HDDS (adjusted OR (95%CI) = 0.29 (0.02, 0.55)). Compared to the data from stage 1, the proportion of people choosing healthy products to cope with COVID-19 did not greatly change and those people had a higher HDDS (adjusted OR (95%CI) = 0.31 (0.19, 0.42)). Although this study found that the proportion of people who chose to use alcohol or vinegar to prevent COVID-19 had decreased substantially compared to during lockdown, there were still 5.3% and 9.8% who followed these irrational behaviors. Regarding the dietary behavior regarding food safety, except for cooking food fully, fewer than half of participants followed the recommended dietary behaviors, including individual food servings (44.2%), using serving chopsticks and spoons (44.8%), and preparing raw and cooked food separately (43.3%). People who followed these behaviors had a better dietary diversity. In conclusion, during the post-lockdown period, people still followed certain dietary behaviors to cope with COVID-19. While some dietary behaviors were adopted to help prevent infection, irrational dietary behaviors were still followed. These behaviors were associated with the dietary diversity in Chinese adults.

## 1. Introduction

Declared as a pandemic by the World Health Organization (WHO), coronavirus disease 2019 (COVID-19) imposes enormous challenges on health systems, the economy, and food supplies globally, and has a great influence on individuals’ lifestyles [1,2]. Chinese residents living both in and outside Hubei had been required to stay at home to self-isolate since 23 January 2020 [3]. After 76 days, on April 8, Wuhan reopened transportation connections to areas outside the city, which marked the end of the lockdown in most parts of China [3]. However, relapses of the domestic COVID-19 epidemic occasionally occurred in some places, such as Beijing, Xinjiang, and Ruili (Yunnan province) [4,5,6]. In the protracted COVID-19 epidemic, certain behaviors were still recommended to residents to prevent infection and maintain health, such as keeping social distancing, wearing masks, and following certain dietary behaviors [7].

In the lockdown time, we conducted the first stage of a nutrition survey on Chinese residents. Based on the data from the first stage of the survey and studies performed outside China during the pandemic, we found people’s dietary behaviors to be greatly affected by COVID-19. Some people chose to eat more snacks and other high-calorie foods to cope with the depression and anxiety triggered by the pandemic, and some intended to prevent coronavirus through drinking alcohol and vinegar [8,9,10,11,12]. The ways to purchase food were also changed by the restriction of outside activities and transportation [8]. These changes in dietary behaviors more or less affected people’s health. Although according to the data from the first stage of the survey, the dietary diversity was generally good [8], the inappropriate dietary behaviors may still raise several nutritional concerns. One previous study reported that the increased intake of high-calorie food contributed to a significant gestational weight gain in women [13]. Iran reported there were at least 2197 people poisoned and 244 deaths due to drinking toxic alcohol (methanol-based beverages) that was believed to prevent COVID-19 [14].

Currently, people in China have gradually returned to normal work and daily life. It is interesting to know whether the dietary behaviors of Chinese people are still affected by COVID-19 and its potential effects on health. In addition, to prevent COVID-19 infection, certain dietary behaviors were encouraged for the Chinese people that are significantly different from those expressed in Chinese culture, such as using serving chopsticks and spoons and serving food individually [15]. Meanwhile, because of the newly diagnosed COVID-19 cases in Beijing being linked to the market and the frozen food sold in the market [16], some professionals suggested that there is a potential risk of infection associated with exposure to frozen food [17]. How these recommendations and suggestions could influence people’s dietary behaviors and further affect the nutrition status in post-lockdown times needed to be explored.

In order to observe the changes in dietary behavior of Chinese residents during the COVID-19 pandemic and to explore the potential dietary behavior that could affect health, the second stage of a longitudinal online nutrition study was conducted in August in China. Data on dietary behaviors and dietary diversity were both investigated and compared with the results of the first stage of the survey conducted during the lockdown.

## 2. Materials and Methods

### 2.1. Study Design and Participants

This paper describes the second stage of a longitudinal nutrition study during the COVID-19 pandemic in China. The first stage was conducted in March 2020 during the lockdown. The details of the methodologies used in the first stage of the survey were described previously [8]. The second stage was conducted in August 2020 (during the post-lockdown period) through a Wenjuanxing e-questionnaire platform (Wenjuanxing Tech Co. Ltd, Changsha, China), which is widely used in China. A multistage sampling method was applied. We purposely selected persons living in northern, southern, and median parts of China. More participants were then reached using a “snowball sampling” method.

The e-questionnaire was logistically set to ensure all the participants were aged from 18 to 80 years, and there were no missing data on key questions. A total of 2267 Chinese residents responded to the survey. After examining all the collected data, those living outside mainland China (*N* = 9) were excluded. In addition, two questions were used in the questionnaire to help identify whether participants had seriously answered the questions or not. One question was placed at the end of the survey, which was: “Have you answered the questions seriously?”. Another was hidden in the middle of the questionnaire: “Please choose the option “very” for this question.” Those who chose “no” in the first question and did not answer “very” to the second question were considered as not responding seriously to the survey and were excluded from the data analysis (*N* = 264). Participants could get a monetary reward after the quality of response was checked by investigators.

**Figure 1:**
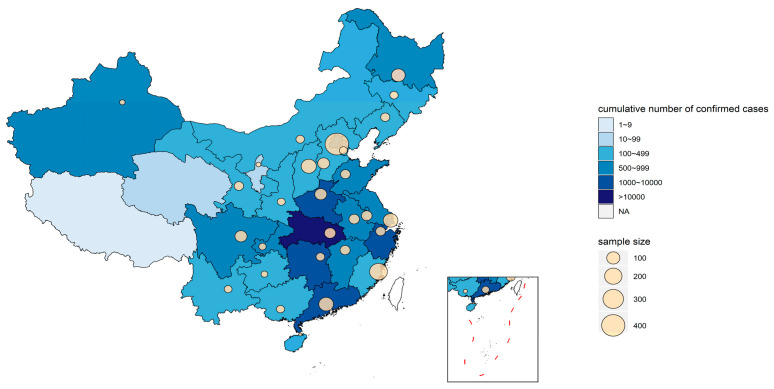
Geographical distribution of the studied population in the second nutrition survey wave. Figure legends: The color of the map indicates the cumulative number of confirmed cases in each area by the end of August 31, according to the report from the Chinese Health Commission [16]. A bubble plot was then generated, with the bubble size representing the number of participants at each investigation point.

Finally, the data of 1994 participants in the second stage of the survey were used in this study. A total of 24.2% of the participants that took part in the second stage of the survey also took part in the first stage [8]. The geographic distribution of the participants in stage two can be seen in Figure 1.

### 2.2. Data Collection

There were four parts to the questionnaire: socio-demographic characteristics, COVID-19 prevalence status in different areas, dietary diversity, and dietary behaviors.

The data of the COVID-19 prevalence status, including the numbers of confirmed cases (the imported cases were not excluded) in each province, autonomous region, or municipality directly under the central government by the end of August and whether the participants’ living areas had a second outbreak, were obtained from the website of the Chinese Health Commission [18].

The dietary diversity was evaluated using the Household Dietary Diversity Score (HDDS), which was developed to measure a household’s access to food [8]. The consumption of 12 food items over the previous 24 h was investigated, including cereals; roots and tubers; vegetables; fruit; meat, poultry, and offal; eggs; fish and seafood; pulses, legumes, and nuts; dairy products; oils and fats; sugar and honey; miscellaneous (such as condiments, snacks, and beverages). Participants were assigned “one” or “zero” points for each food item depending on whether or not they had consumed it in the previous 24 h. The HDDS total ranges from 0 to 12.

A series of questions regarding changes in dietary behaviors were included. The studied population was required to self-report whether they showed increased, unchanged, or decreased frequencies of certain dietary behaviors compared to the same period in 2019, such as eating outside, cooking at home, consumption of seafood, and consumption of imported frozen food. In line with the first stage of the survey, participants were asked whether they currently had specific dietary behaviors to cope with COVID-19, including intentionally taking dietary supplements (vitamin C, probiotics, and other dietary supplements), functional food (such as ginseng and Ganoderma), and food (including vinegar and alcohol). Whether the participants followed the updated recommended behaviors after the COVID-19 outbreak were also investigated, including individual food servings, using serving chopsticks and spoons, fully cooking food, and preparing raw and cooked food separately.

To investigate food purchasing sources, consistent with questions used in the first stage of the survey, four common approaches to obtaining or purchasing different types of foods (the 12 food items in the HDDS) were asked about, namely, (1) in-house storage, (2) in-person grocery shopping, (3) using online food ordering and delivery services (including purchasing both raw ingredients and prepared meals from restaurants), and (4) being dependent on government- or community-based food distribution.

### 2.3. Ethics

The questionnaire was filled in anonymously. Informed consent was required prior to the survey by clicking the “agree” option to confirm the willingness to participate voluntarily in the survey.

### 2.4. Statistics

Data were analyzed using SAS v9.4 (SAS Institute, Cary, NC, USA). Data were presented as mean ± standard deviation (SD) or a percentage. The comparisons of the HDDSs for participants with different characteristics were tested with an independent *t*-test or ANOVA analysis.

A linear regression model was used to explore the associations of participants’ dietary behaviors with the HDDS after adjusting for the family income and living in an area with a second outbreak of COVID-19. Regarding the food purchasing pattern, the clustering process was conducted with the flexclust package within R 4.0.2. (R Core Team, Vienna, Austria) We first ran a K-means cluster analysis on the data collected in the first stage and identified three patterns of food-collecting propensity. Subsequently, we ran the “predict()” function to cluster the data collected in the second stage using the centroids from the previous analysis. Finally, three food purchasing patterns were explored and the differences in the HDDSs between the three different patterns were examined. A *p*-value < 0.05 was considered statistically significant in all analyses.

### 2.5. Heat Map

The heat map of the geographical distribution of participants were created using R v3.6.3 (R Core Team, Vienna, Austria) with the packages “ggplot2,” “maptools,” “rgdal,” and “sp.” The base map was obtained as a shape file from the National Fundamental Geographic Information System, China. The cumulative number of confirmed cases of COVID-19 were obtained at the province, autonomous region, or municipality directly under the central government level and identified with different colors, where the darker the color, the more cases. A bubble plot was then generated, in which the bubble size represented the number of studied samples.

## 3. Results

### 3.1. The HDDSs of Participants with Different Characteristics

A total of 1994 adults participated in this study, where 483 (24.2%) had also been enrolled in stage 1 of the nutrition survey. Comparing the socio-demographic characteristics of participants in stages 1 and 2 showed the participants in stage 2 were relatively young, living in urban areas, and with both higher rates of lower (senior high school or under) and higher (master’s degree or above) education levels (Table S1).

The average HDDS was 9.2 ± 2.0. During the post-lockdown period, people with a higher family income showed a significant higher HDDS (Table 1). Other socio-demographic characteristics were not associated with the HDDS. The calculated number of confirmed cases of COVID-19 (≥500 or <500) and the occurrence of a second outbreak of COVID-19 were not associated with the HDDS.

### 3.2. Dietary Behaviors and Their Association with the HDDS

A substantial proportion of participants self-reported that they recently decreased their eating at restaurants (61.6%) and reduced their intake of seafood (53.1%), imported frozen food (57.1%), and raw food (60.5%), while 64.8% of participants reported increased their amount of cooking at home in comparison to the same period in the previous year (2019).

In the univariate analysis, the people who increased their consumption of seafood and raw food had a significantly higher HDDS. Participants who changed their consumption of imported frozen food (both increased and decreased) had a higher HDDS (Table 2).

Based on a K-means clustering analysis, the studied population was clustered into three groups. People in cluster 1 showed a higher dependence on in-person grocery shopping for food, people in cluster 2 depended more on both in-person grocery shopping and in-house storage, and people in cluster 3 depended mostly on online food ordering and delivery. There were 1060 (53.2%), 649 (32.5%), and 285 (14.3%) participants in clusters 1, 2, and 3, respectively. People in cluster 3 showed a significantly higher HDDS (9.5 ± 2.0) compared with people in clusters 1 (9.2 ± 1.9) and 2 (9.1 ± 2.0), (*p* = 0.021).

Regarding the dietary behaviors used to cope with COVID-19 in the post-lockdown time, there was a total of 661 (33.1%) participants who used dietary supplements (vitamin C, probiotics, and other dietary supplements) and functional food (such as ginseng and Ganoderma) to cope with COVID-19, and a small proportion of the studied participants still used drinking alcohol and vinegar. Participants who chose to take vitamin C, probiotics, other dietary supplements, and functional food to cope with COVID-19 had a significant higher HDDS (Table 3).

Regarding the recommended dietary behaviors, in the post-lockdown time, 44.2%, 44.8%, 93.8%, and 43.3% of participants who followed individual food serving, using serving chopsticks and spoons, cooking food fully, and preparing raw and cooked food separately recommendations, respectively. People who used serving chopsticks and spoons and prepared raw and cooked food separately had a significantly higher HDDS (Table 4).

### 3.3. Multivariable Analysis of the Dietary Behaviors Associated with the HDDS

As part of the multivariable analysis (Table 5), after adjusting for family income and living areas (whether the areas had a second outbreak), the increased online food purchasing, increased consumption of seafood, both increased and decreased consumption of imported frozen food, increased intake of raw food, and intake of dietary supplements (including vitamin C, probiotics, and other supplements and health products) were associated with a higher HDDS. People who did not follow the behaviors of using serving chopsticks and spoons or preparing raw and cooked food separately had a lower HDDS.

## 4. Discussion

With the subsequent development of a global pandemic, COVID-19 has significantly changed the lifestyles and behaviors of people temporarily and may also bring permanent effects. Comparing data with that from the first stage of the survey that took place during the lockdown, the current second stage of the study showed that people still followed certain dietary behaviors to cope with COVID-19. Meanwhile, new dietary behaviors were acquired to help prevent infection. These behaviors were directly associated with the dietary diversity in Chinese adults.

### 4.1. Dietary Behavior Changes Compared with 2019

The WHO has specifically said that there is no evidence of food having been a source of infection during the pandemic, either through eating or touching contaminated food [19]. At the same time, however, some recent outbreaks of COVID-19 were reported to be closely related to food or markets [16,20,21], and many countries, including China, had reported finding traces of the virus on packages of frozen food in the market [22,23]. Therefore, it is not difficult to see that, compared with the same period in 2019, this study showed more than half of participants reported that they decreased their eating at restaurants (61.6%) and their consumption of seafood (53.1%), as well as importing frozen food (57.1%) and raw food (60.5%), and at the same time, more people preferred to choose self-cooking at home (64.8%). One newly released preprint on the biorxiv website shows that the coronavirus can survive on the surface of frozen food for up to three months, which indicates food-borne viruses are at least theoretically possible [24]. In this case, on the one hand, Chinese residents spontaneously reduced their intake of raw and frozen food, the frequency of eating outside, and the consumption of takeaways. On the other hand, many places removed frozen food from market storage, especially imported frozen seafood, which greatly impaired food accessibility [25]. It is also worth noting that an insufficient intake of seafood in the Chinese population had also been observed in a previous study conducted before the COVID-19 pandemic and during lockdown time [8,26]. The exacerbating decreased intake of seafood may bring potential nutrition concerns, especially for the ones who were living in inland areas, where most of their fish and seafood consumption depended on frozen food.

### 4.2. Dietary Behavior Changes Compared with the Lockdown Period in the COVID-19 Pandemic

When comparing the behaviors in the post-lockdown period with those during the lockdown, we found that the rate of using dietary supplements and functional food to cope with COVID-19 was not greatly changed in participants between stage 1 (37.7%) and stage 2 (33.1%) [8]. It is worth noting that irrational and unhealthy dietary behaviors still existed in a small proportion of participants. Although there was a great decline in people who chose to drink alcohol and vinegar to cope with COVID-19 compared with stage 1 [8], there were still 5.3% and 9.8% of participants, respectively, doing these behaviors in the post-lockdown period. The Chinese government had already dismissed the rumor that drinking alcohol or vinegar could prevent coronavirus as early as 22 January 2020 [27]. It seems that health education is still urgently needed and should be continued in any protracted COVID-19 pandemic.

### 4.3. Dietary Behaviors Regarding Food Safety

The dietary behaviors regarding food safety were also investigated. Cooking food fully and preparing raw and cooked food separately were always the key messages in food safety education to avoid food-borne disease [28,29]. In the current study, most participants followed the advice to cook food fully. However, 31.9% of participants did not prepare raw and cooked food separately, which may easily cause food contamination. In addition, according to the lessons learned from COVID-19, China currently encourages the public to use serving chopsticks and spoons and to adopt individual food servings [13]. However, since these behaviors are very different from traditional Chinese customs, this study found that more than half of the participants did not follow these new behaviors. To minimize the risk of COVID-19 infection, changes in dietary behavior regarding food safety should be incorporated in the current work on health promotion.

### 4.4. Food Purchasing Behaviors

With the development of e-commerce, China has become a front-runner in online retail due to it having the largest online population growth in the world; online ordering services have already been integrated into our daily life [30]. The success of China’s fight against the pandemic cannot be separated from the cooperation of the whole nation and citizens’ voluntary isolation at home. Online food services were strong guarantees of food accessibility during the lockdown. As the Global Times reported on the COVID-19 pandemic, online delivery services of fresh food increased 2.5–4-fold during the lockdown in China [31]. Contactless online food shopping by the public was also encouraged in many other countries [32]. In stage 2, 14.3% of participants mostly depended on online food shopping and deliveries. However, based on the currently limited data on online food services in the pandemic, several concerns were raised, such as, in some countries, online food services provided more highly energy-dense food, the coverage of online services could not reach rural areas, vulnerable groups were restricted in their access to digital technology, and there are potential food safety issues [33,34].

### 4.5. Dietary Behaviors Associated with the Diet Diversity

Dietary behaviors could greatly affect people’s dietary intake, nutrition, and health. This study revealed that participants who increased their intake of seafood and raw food (including fruit and vegetables) had a significantly higher HDDS. Although some frozen food and seafood samples were found to be contaminated with the coronavirus, the infection could be avoided by appropriately handling and dealing with the food. According to the U.S. Center for Disease Control and Prevention advice, the risk of infection with COVID-19 could be minimized by following basic steps for food safety [35]. The current survey also observed not only that an increased intake of imported frozen food was associated with a higher HDDS but that a decreased intake was also related to a higher HDDS. We infer that this may be because the decrease in imported frozen food could be replaced by similar local food, and people who decided not to choose imported frozen food may usually pay more attention to their nutrition and health. In addition, this study also demonstrated that people who followed healthier dietary behaviors, including choosing to use dietary supplements and functional food, using serving chopsticks and spoons, and preparing raw and cooked food separately, had a higher HDDS. This could be explained by recognizing that this group of people was more concerned about their health and followed a healthier lifestyle, including eating a diversified diet.

It is worth noting that in stage 1 of our survey, we showed that the online ordering of food could help people to maintain dietary diversity during the lockdown [8]. In the current stage of the survey, we further identified that people who depended mainly on the online purchasing of food during the post-lockdown period also had a better HDDS. Even so, after combining the data from the first and second stages of the survey, there are reasons to believe that under strict supervision and management of food safety, the use of online food services could serve as a flexible way to help people purchase food contactlessly and ensure nutrition in the COVID-19 era.

### 4.6. Limitations

For convenience and in line with the first stage of the survey, the HDDS was used in this study. This is the index that can reflect food accessibility, and to some extent, be used to predict the risk of malnutrition; however, some validation studies mentioned that the HDDS does not provide a reliable reflection of the household-level access to food [36] and it cannot quantitatively assess the sufficient or insufficient intake of nutrients. Based on the online anonymous survey methodology, unfortunately, we could not follow up on all the participants in stage 1; there were 24.2% of participants enrolled in both stages 1 and 2. The socio-demographic characteristics were not the same, where differences were found in age, education level, and geographic living areas. In addition, some vulnerable groups who were not familiar with digital technology could not be reached, and the dietary behaviors of these populations might be very different. Regarding dietary behavior, we did not measure the rate of using serving chopsticks and spoons, individual servings, etc., before and during the lockdown. Although these behaviors are obviously not traditional Chinese customs, we could not estimate exactly how many people picked up these new rules during the COVID-19 pandemic.

## 5. Conclusions

With a longitudinal study design, we observed the changes in dietary behavior between the lockdown and post-lockdown periods. People followed certain behaviors to prevent disease, such as decreasing outside eating; decreasing their consumption of raw food, seafood, and imported frozen food; increasing the frequency of cooking at home. Some unhealthy and irrational behaviors, including drinking alcohol or vinegar to prevent infection, were decreased but still existed. Meanwhile, following recommendations, more than half of the people used serving chopsticks/spoons and adopted individual servings. These behaviors had certain impacts on the dietary diversity. To ensure food diversity, the online food ordering and delivery services were very helpful in both the lockdown and post-lockdown periods.

Based on the results of the current study, education on how to scientifically and safely obtain, purchase, deal with, and eat food is still greatly needed and should be carried out throughout the whole COVID-19 era, even during the post-lockdown period.

## Figures and Tables

**Table 1:** Household Dietary Diversity Scores (HDDSs) of participants with different characteristics.

Characteristics of Participants		*N*	HDDS (Mean ± SD)	*p*
Age	18–45 years old	1778	9.2 ± 2.0	0.533
	>45 years old	216	9.4 ± 1.9	
Gender	Male	742	9.2 ± 1.9	0.743
	Female	1252	9.2 ± 2.0	
Education level	Senior high school or under	389	9.2 ± 2.1	0.528
	Bachelor’s degree	1151	9.2 ± 1.9	
	Master’s degree or above	454	9.1 ± 1.9	
Family annual income (Chinese yuan)	<30 thousand	215	8.6 ± 2.2	<0.001
	30–100 thousand	695	9.1 ± 2.0	
	>100–300 thousand	765	9.3 ± 1.9	
	>300 thousand	319	9.6 ± 1.8	
Geographic region	Urban	1645	9.3 ± 1.9	0.067
	Rural	349	8.8 ± 2.1	
Vulnerable person living in the house ^a^	Yes	13	10.3 ± 1.9	0.752
	No	1981	9.2 ± 2.0	
Confirmed cases in the province	<500	682	9.2 ± 2.0	0.453
	≥500	1312	9.2 ± 1.9	
Second COVID-19 outbreak	Yes	669	9.2 ± 2.0	0.932
	No	1325	9.2 ± 1.9	

^a^ The vulnerable people were children under 5 years old, elders above 65 years old, and pregnant and lactating women.

**Table 2:** Comparison of the HDDSs of participants with different behaviors.

Behaviors Comparing with 2019		*N*	HDDS (Mean ± SD)	*p*
Eating at restaurants	Increased	247	9.2 ± 2.1	0.907
	Stay the same	519	9.2 ± 2.0	
	Decreased	1228	9.2 ± 1.9	
Eating takeaways	Increased	420	9.3 ± 2.1	0.469
	Stay the same	583	9.1 ± 2.0	
	Decreased	991	9.2 ± 1.9	
Cooking at home	Increased	1292	9.3 ± 1.9	0.286
	Stay the same	514	9.1 ± 2.0	
	Decreased	188	9.1 ± 2.2	
Purchasing food from a market	Increased	599	9.3 ± 1.9	0.623
	Stay the same	727	9.2 ± 1.9	
	Decreased	668	9.2 ± 2.0	
Purchasing food online	Increased	824	9.4 ± 1.9	<0.001
	Stay the same	633	9.1 ± 2.0	
	Decreased	537	9.0 ± 2.1	
Consuming seafood	Increased	189	9.6 ± 1.9	0.004
	Stay the same	747	9.2 ± 1.9	
	Decreased	1058	9.1 ± 2.0	
Consuming frozen food	Increased	377	9.3 ± 1.9	0.396
	Stay the same	799	9.1 ± 2.0	
	Decreased	818	9.2 ± 2.0	
Consuming imported frozen food	Increased	67	9.7 ± 2.1	0.002
	Stay the same	788	9.0 ± 1.9	
	Decreased	1139	9.3 ± 2.0	
Consuming raw food	Increased	69	9.9 ± 2.2	0.005
	Stay the same	718	9.1 ± 1.9	
	Decreased	1207	9.2 ± 2.0	
Consuming snacks and beverages	Increased	497	9.2 ± 2.0	0.587
	Stay the same	749	9.2 ± 2.0	
	Decreased	748	9.1 ± 2.0	

**Table 3:** Comparison of the HDDSs between participants with different coping behaviors against COVID-19 in the post-lockdown time.

Behaviors to Cope with COVID-19		*N* (%)	HDDS (Mean ± SD)	*p*
Intake of vitamin C	Yes	503 (25.2)	9.5 ± 1.9	<0.001
	No	1491 (74.8)	9.1 ± 2.0	
Intake of a probiotic	Yes	257 (12.9)	9.6 ± 2.0	0.001
	No	1737 (87.1)	9.1 ± 1.9	
Intake of other health products	Yes	166 (8.3)	9.7 ± 1.9	<0.001
	No	1828 (91.7)	9.2 ± 2.0	
Intake of alcohol	Yes	105 (5.3)	9.5 ± 2.3	0.078
	No	1889 (94.7)	9.2 ± 1.9	
Intake of vinegar	Yes	196 (9.8)	9.5 ± 2.1	0.057
	No	1798 (90.2)	9.2 ± 1.9	

**Table 4:** Comparison of the HDDSs of participants with different behaviors regarding food safety.

Behaviors Regarding food Safety		*N* (%)	HDDS (Mean ± SD)	*p*
Individual food servings	Yes	882 (44.2)	9.2 ± 2.0	0.386
	No	1112 (55.8)	9.2 ± 1.9	
Using serving chopsticks and spoons	Yes	894 (44.8)	9.3 ± 2.0	0.006
	No	1100 (55.2)	9.1 ± 1.9	
Fully cooked food	Yes	1930 (96.8)	9.2 ± 1.9	0.084
	No	64 (3.2)	8.8 ± 2.2	
Preparing raw and cooked food separately	Yes	1357 (68.1)	9.4 ± 1.9	<0.001
	No	637 (31.9)	8.8 ± 2.0	

**Table 5:** Multivariable analysis of dietary behaviors associated with the HDDS.

Behaviors		HDDS			
		Crude *β* (95%CI)	*p*	Adjusted-*β* (95%CI) ^a^	*p*
Dietary behavior changes compared with the same period in 2019					
Consuming seafood ^a^	Increased	0.45 (0.15, 0.76)	0.004	0.39 (0.09, 0.70)	0.010
	Stay the same	Reference		Reference	
	Decreased	−0.05 (−0.24, 0.13)	0.567	−0.00 (−0.19, 0.18)	0.962
Consuming imported frozen food ^a^	Increased	0.67 (0.18, 1.15)	0.008	0.56 (0.07, 1.04)	0.026
	Stay the same	Reference		Reference	
	Decreased	0.26 (0.08, 0.44)	0.004	0.27 (0.09, 0.44)	0.003
Consuming raw food ^a^	Increased	0.79 (0.32, 1.27)	0.001	0.74 (0.27, 1.21)	0.002
	Stay the same	Reference		Reference	
	Decreased	0.11 (−0.07, 0.29)	0.244	0.08 (−0.10, 0.26)	0.359
Food-purchasing behaviors					
Food purchasing methods ^b^	Cluster 1	Reference		Reference	
	Cluster 2	−0.26 (−0.22, 0.16)	0.786	−0.01 (−0.20, 0.18)	0.940
	Cluster 3	0.40 (0.14, 066)		0.29 (0.02, 0.55)	0.035
Dietary behaviors used to cope with COVID-19 in the post-lockdown time					
Intake of health products ^c^	Yes	0.39 (0.21, 0.57)	<0.001	0.31 (0.19, 0.42)	<0.001
	No	Reference		Reference	
Recommended dietary behaviors to prevent foodborne disease					
Using serving chopsticks and spoons	Yes	Reference		Reference	
	No	−0.24 (−0.41, −0.07)	0.006	−0.29 (−0.46, −0.11)	0.001
Preparing raw and cooked food separately	Yes	Reference		Reference	
	No	−0.54 (−0.73, −0.36)	<0.001	−0.52 (−0.70, −0.34)	<0.001

^a^ Adjusted for family income and living in areas where a second outbreak episode occurred. ^b^ People in cluster 1 showed a higher dependence on in-person grocery shopping for food, people in cluster 2 depended more on both in-person grocery shopping and in-house storage, and people in cluster 3 depended mostly on online food ordering and deliveries. ^c^ The health products included vitamin C, probiotics, and other dietary supplements.

## Supplementary Materials

The following are available online at https://www.mdpi.com/2072-6643/12/11/3269/s1, Table S1: Socio-demographic characteristics of participants in stages 1 and 2 of the nutritional survey.
