# Supplementary material for: Dietary Behaviors in the Post-Lockdown Period and Its Effects on Dietary Diversity: The Second Stage of a Nutrition Survey in a Longitudinal Chinese Study in the COVID-19 Era
Source: Nutrients. 2020 Oct 26;12(11):3269. doi: 10.3390/nu12113269 (PMC7693097; doi:10.3390/nu12113269)
Supplement: Supplementary file 1 [file nutrients-12-03269-s001.pdf]

Supplementary table 1. Socio-demographic characteristics of participants in the 1 and 2 stage of nutritional survey

| | | Stage 1 N=1938 | Stage2 N=1994 | P |
|---|---|---|---|---|
| **Age** | 18-45y | 1620 （83.6） | 1778（89.2） | <0. 001 |
| | >45y | 318（16.4） | 216（10.8） | |
| Gender | Male | 665(34.3) | 742(37.2) | 0.061 |
| | Female | 1273(65.7) | 1252(62.8) | |
| Education level | Senior high school or under | 219(11.3) | 389(19.5) | <0. 001 |
| | Bachelor's degree | 1464(75.5) | 1151(57.7) | |
| | Master's degree or above | 255(13.2) | 454(22.8) | |
| Family annual income (Chinese yuan) | <30 thousand | 206(10.6) | 215 | 0.860 |
| | 30-100 thousand | 690(35.6) | 695 | |
| | >100-300 thousand | 750(38.7) | 765 | |
| | >300thousand | 292(15.1) | 319 | |
| Geographic Region | Urban | 414(21.4) | 349(17.5) | <0. 001 |
| | Rural | 1524(78.6) | 1645(82.5) | |
| Vulnerable group living in the house[a] | Yes | 10(0.5) | 13(0.7) | 0.576 |
| | No | 1928(99.5) | 1981(99.3) | |
| | ≥500 | 862(44.5) | 1312(65.8) | |

Nutritional survey of stage 1 and 2 were respectively carried out at March(lockdown period) and August(post-lockdown period), 2020.

a. Vulnerable group indicates children under 5y, elders above 65, pregnant and lactating women.
